# Highly Effective Photonic Cue for Repulsive Axonal Guidance

**DOI:** 10.1371/journal.pone.0086292

**Published:** 2014-04-09

**Authors:** Bryan J. Black, Ling Gu, Samarendra K. Mohanty

**Affiliations:** Biophysics and Physiology Group, Department of Physics, The University of Texas at Arlington, Arlington, Texas, United States of America; The University of Akron, United States of America

## Abstract

In vivo nerve repair requires not only the ability to regenerate damaged axons, but most importantly, the ability to guide developing or regenerating axons along paths that will result in functional connections. Furthermore, basic studies in neuroscience and neuro-electronic interface design require the ability to construct *in vitro* neural circuitry. Both these applications require the development of a noninvasive, highly effective tool for axonal growth-cone guidance. To date, a myriad of technologies have been introduced based on chemical, electrical, mechanical, and hybrid approaches (such as electro-chemical, optofluidic flow and photo-chemical methods). These methods are either lacking in desired spatial and temporal selectivity or require the introduction of invasive external factors. Within the last fifteen years however, several attractive guidance cues have been developed using purely light based cues to achieve axonal guidance. Here, we report a novel, purely optical repulsive guidance technique that uses low power, near infrared light, and demonstrates the guidance of primary goldfish retinal ganglion cell axons through turns of up to 120 degrees and over distances of ∼90 µm.

## Introduction

It is well known that axonal pathfinding [1], [2] is paramount to an organism's nervous system development [3], [4], and that, during this development, functional connections must be made across the entire organism [5], [6], [7]. Furthermore, *in vivo* nerve repair (e.g., following spinal cord injury [8]) requires not only the ability to regenerate damaged axons, but, most importantly, the ability to guide these regenerating axons along paths that will again result in functional connections. Basic studies in neuroscience and design of effective neuro-electronic interface devices [9] require the ability to construct *in vitro* neural circuitry [10]. Therefore, the ability to effectively guide single axons is important to the understanding of basic, as well as clinical, problems. It has been shown that axonal growth rates and direction are primarily determined by environmental cues [11] which are ‘sensed’ by the axon's filopodia [1]. These filopodia are finger-like growth cone extensions which sample the surroundings for attractive or repulsive growth and guidance cues, which may be mechanical, electrical, or chemical in nature. These cues can effectively induce or inhibit axonal growth [12] by affecting actin filament polymerization processes. If such gradients are asymmetrically positioned along the axonal growth axis, they result in growth cone turning and ultimately short-to-long-range axonal guidance. This has been explained by observations that a growth cone's filopodia react independently to these guidance cues (even independently from the growth cone itself) [1]. For example, activation of a filopodium's calcium ion channels can result in an influx of extracellular calcium, which causes actin depolymerization, resulting in the arrestment of a filopodium's growth, or even complete retraction into the axon's lamellipodium (growth cone) [13]. This has been the basis for several axonal guidance [14] techniques introduced over the past two decades. It has been shown that chemical [15], topographical [16], electrical [17], and hybrid approaches such as electro-chemical [18], optofluidic flow [19] and photo-chemical [20] methods can guide neuronal growth cones *in vitro*. However, these methods are either lacking in spatial and/or temporal specificity, or require the introduction of external factors. Therefore, the development of selective, minimally-invasive methods of axonal guidance is of considerable importance in the fields of neuroscience and neuroengineering.

To date, all purely optical methods (optical tweezers [21], [22], [23], asymmetric line tweezers [24], tapered optical fiber [25], [26], and ultrafast laser microbeam [27], [28]) have been employed as an attractive axonal guidance cue. These methods use a focused laser beam operated in continuous wave (cw) or pulsed mode in order to utilize the intensity/force gradient or for microsurgery [28] (at the highest intensity region of the beam center). The guidance mechanisms in these cases rely on a tightly focused laser beam (high NA microscope objective with short working distance) impinging directly upon an individual growth cone. Here, we show that asymmetrical positioning of a laser beam in front of an advancing growth cone produces significant repulsive guidance. This repulsive optical cue leads to highly efficient and long-range guidance of goldfish retinal ganglion cell (RGC) axons. We also theoretically explore the plausible mechanisms for the optical guidance effect and effectively rule out physical forcing or activation of stretch-sensitive ion channels. The presented method could allow efficient construction of *in vitro* neural circuitry for study of functional aspects of basic neuroscience and also paves the way for controlling the path of *in vivo* nerve regeneration.

## Materials and Methods

### Ethics statement

All experimental procedures were conducted according to the University of Texas at Arlington-Institutional Animal Care and Use Committee approved protocol A10.010.

### Retinal explant preparation

Retinal ganglion cells of common goldfish, *Carassius auratus*, were used in all experiments. Adult fish, 5–7 cm in body length, were housed in a standard glass aquarium at 19°–21°C. In order to facilitate axonal outgrowth in culture, fish received a priming lesion of the optic nerve at least 1 week before removing the retina [29], [30]. Following anesthesia with tricaine methanesulfonate (Argent), the optic nerve was visualized through an incision in dorsal-posterior conjunctiva and crushed with Dumont #5 forceps distally where the nerve exits the orbit. Care was taken to avoid damaging the large blood vessel which runs alongside the nerve. One to 2 weeks post-crush, eyes were removed from the anesthetized goldfish after one hour of dark adaptation to facilitate removal of the pigment epithelium. The retinas were removed and cut into 400 µm square explants on a Mcllwain tissue chopper. The explants were then placed into sterile 35-mm Petri dishes with a 14 mm central hole backed by a glass coverslip (MatTek) previously coated with 0.75 mg/dish poly-D-lysine (Sigma, >300,000 MW in borate buffer, pH 8.3) and 5 µg/dish of laminin (BD Biosciences, in phosphate-buffered saline, pH 7.4). The explants were oriented ganglion cell side toward the laminin, and incubated at room air and temperature in Leibovitz's L15 medium (Sigma) supplemented with 10% fetal bovine serum (Fisher Scientific) and 50 µg/ml gentamicin. Since goldfish are poikilothermic, it is easy to maintain the retina in the Petri dishes at room temperature and visualize neuronal growth under the microscope.

### Axonal navigation and analysis

The schematic of the imaging and microscopic manipulation platform for axonal navigation experiments is shown in Figure S1a in File S1. Repulsive optical guidance of healthily advancing growth cones was achieved with a tunable Ti: Sapphire laser (MaiTai HP, Newport-SpectraPhysics) operated in mode-lock off condition to ensure cw-mode usage. The near-infrared laser beam was expanded and relayed via folding mirrors to the back-laser port of an inverted microscope (Ti-U Eclipse, Nikon). A mechanical shutter (Uniblitz) was used continuously throughout each experiment to pulse the laser beam (200 ms ON and 200 ms OFF) in order to avoid continuous mechanical forcing. A dichroic mirror was used to guide the beam to the back-aperture of a microscope objective (Nikon 100×, NA = 1.3). An IR cut-off filter was used in the imaging path for removing the reflected laser beam from the images collected by the EMCCD (Photometrics). For wavelength dependent experiments, the power of the laser beam was adjusted via the MaiTai control software and external polarizer so as to result in a sample-site beam power of ∼50 mW. In order to achieve repulsive optical guidance, the laser beam spot was asymmetrically positioned in the path of the advancing axons and left static for the duration of the experiment unless otherwise noted.

All image sequences were collected and processed using ImageJ software (NIH). In order to determine the turning angle, ImageJ's angle tool was used, with the vertex for each measurement kept approximately 5 µm from the leading edge of the advancing growth cone. Growth rates and distances determined by tracing distances from a fixed arbitrary point along the axon shaft to the distal end of the advancing growth cone. Axons which did not advance beyond the static position of the laser spot during guidance trials were omitted from statistical comparison with controls.

## Results and Discussion

### Highly efficient optical guidance of axons using laser as repulsive cue

**Figure 1:**
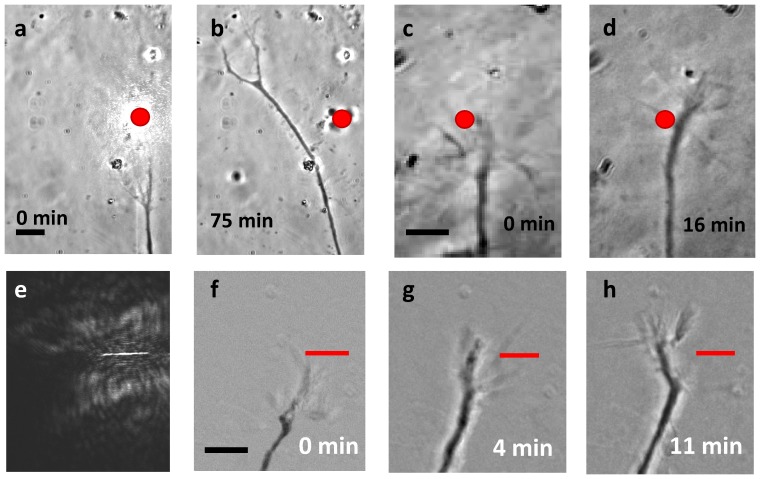
Optically controlled axonal guidance. Left-turning event before (a) and after (b). Right-turn before (c) and after (d). Static laser spot position is indicated by the red dot. Spatially-sculpted light for optical guidance: (e) line-spot profile, (f–h) Time-lapse images of axonal guidance using spatially-sculpted line beam profile (marked as red line). Scale bars represent 10 µm.

For optical guidance of axons, *in vitro* experiments were conducted on goldfish RGC axons emerging from retina explants in petridish. Figure S1a in File S1 shows the schematic for our imaging and optical manipulation platform used in all axonal guidance experiments. The laser beam spot, acting as a repulsive cue, was asymmetrically positioned in front of randomly-selected, advancing axons. Unless otherwise indicated, the laser spot was not repositioned during the course of the experiment. The axons could be selectively turned either “right” or “left,” with turning direction determined by left/right asymmetric placement of the static laser spot. Fig. 1a shows the laser spot (average laser power: 50 mW, 25 Hz), placed in the right-forward position of an advancing growth cone. The axon was observed to turn left (away from the laser spot) by more than 40° (Fig. 1 a to b). Axons could also be turned to the right (angle >30°) (Fig. 1c to d) as a consequence of placing the laser spot in the left-forward position with respect to the advancing growth cone. Figure S1b in File S1 shows a pseudo-colored time-lapse overlay of growth cone turning due to the repulsive nature of the laser spot. In all cases, the axonal shaft was found to eventually realign itself along this new growth cone direction. This may be attributed to the fact that the flexural rigidity [31] of microtubule bundles (2×10^−23^ Nm^2^), located within axonal shafts, is high enough not to allow sharp turns (>15°) over time. Furthermore, the dynamics of actin-based motility prefer growth along relaxed, straight paths, as opposed to stressed turns. All phantom-spot (no laser) trials resulted in the growth cone passing through, or very near to, the phantom laser spot, with negligible growth cone turning (5.55±3.42, 20 min trials, n = 8). Comparison of axon migration paths subject to optical guidance and phantom laser spot trials using a one-tailed t-test yields a p-value of 2.7E-5. The turns achieved by repulsive optical guidance are statistically significant. Figure S2 in File S1 shows a time-lapse sequence of a phantom-spot experiment.

The efficiency of our optical guidance method is found to be 100% (n = 21). For efficacy measurements, we define a complete axonal guidance trial as an experiment during which the axon's growth cone ultimately extended beyond the static position of the laser spot. Axons which were not observed to advance beyond the position of the static laser spot were omitted from statistical analysis. We reiterate that no growth cones were found to successfully advance through the laser spot with the above-mentioned laser parameters. It may be noted that the irradiation schedule in earlier cw laser based attractive-cue based guidance methods (scanning, defocused or spatially-sculpted optical tweezers) rely on the laser spot impinging on the growth cone itself. Here, in contrast to direct irradiation, the laser spot is placed asymmetrically ahead of the actively growing growth cone (Fig. 1 and Figure S1b in File S1), and serves as a highly-effective repulsive cue.

### Spatially-sculpted light for axonal guidance

In some cases (n = 2), during single spot-illumination based optical guidance, the leading asymmetric placement did not correlate to oppositely directed guidance due to initial growth cone retraction from the laser spot. Following retraction, the growth cone was observed to “feel out” all new available paths before again advancing with a defined direction, sometimes generating the unexpected turning direction. However, this effect could be corrected in the future by alternative beam profiles, multiple laser spots, or dynamic control of the laser spot. In order to better define the guidance path, a line beam profile (Fig. 1e) was generated by a cylindrical lens, which could be rotated to define the orientation of the profile. Axonal guidance using the line beam profile is shown in Fig. 1 (f to h) achieved using 785 nm laser beam. Since the laser power is distributed over a relatively large spatial scale (5 µm), higher laser power (200 mW) was used for these experiments. It is worth noting that a spatial light modulator (SLM) can be used to define more complex paths for the migrating axons. With time-sharing scanning laser beams, lower power levels can be used to create similar effects.

### Kinetics of laser-assisted axonal turning

**Figure 2:**
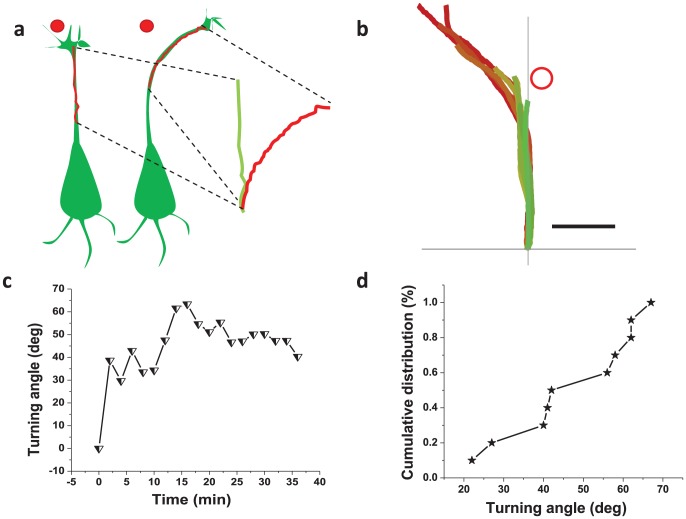
Optically-repulsive axonal guidance is highly effective. (a) Illustration of the method employed for determining efficacy of optical guidance. Laser spot position (red circle). (b) Overlay of time-lapse images of axonal shaft turning toward left. Laser spot position is indicated by red circle. Scale bar represents 5 µm. (c) Kinetics of axonal left-turning angle. (d) Cumulative percentage plots for the distribution of final axon turning angles.

For determining the efficacy of optical beam in axonal guidance over a period of time, we tracked a length of the axonal shaft (starting from growth cone toward soma) and overlaid the tracks as illustrated in Fig. 2a. Fig. 2b shows overlay of time-lapse images of axonal shaft turning toward left due to right-positioning of the laser spot. The kinetics of axonal left-turning angle is shown in Fig. 2c. As shown, the turning process is faster during the initiation phase (5–10 min), which saturates after 15 min. This behavior is quite similar to that observed during right-turning of axons. In Figure S3a in File S1 and Figure S3b in File S1, we show overlay of time-lapse images of axonal shaft turning toward right and kinetics of turning angle, respectively. Fig. 2d shows a cumulative distribution plot for final growth cone turning angles. The majority (8 out of 10) of experiments resulted in growth cone turning angles equal or greater to 42°, while the remaining (2 out of 10) axons turned >22° in response to laser spot. The cumulative distribution of turning in both left and right directions is shown in Figure S4a in File S1. Advancing growth cones were turned by an average of 51.1±14.2° from their initial paths by single laser spot based optical guidance. Prior to laser spot interaction, the observed growth rate was 0.73±0.84 µm/min, well within the range observed in earlier studies [32] [19] [33]. After the growth cone had turned and was no longer interacting with the laser spot, the observed growth rate was measured to be 0.79±0.98 µm/min, illustrating that the guidance event is not damaging in the long term. Growth kinetics of a typical axon is shown in Figure S4b in File S1. This implies that this guidance method is not a permanently damaging event and can be used for guidance and delivery of healthy axons. It is also clear that the induced repulsive turn is a permanent event.

### Long-range optical guidance of axon

**Figure 3:**
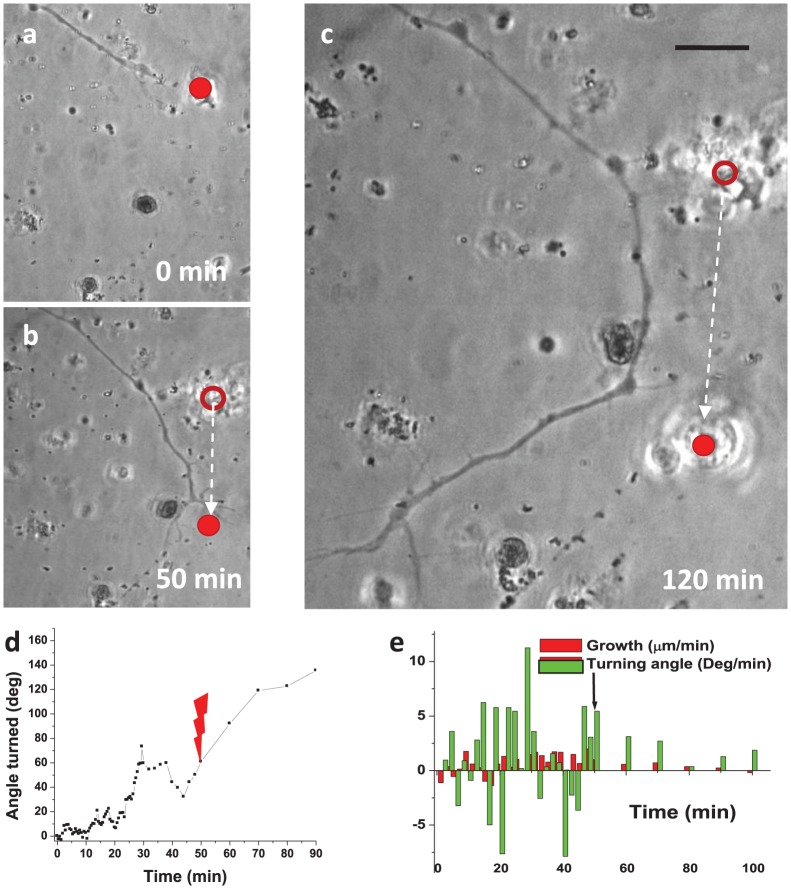
Repulsive long-range axonal guidance using dynamically-positioned laser beam. (a–c) Time-lapse images of long-range optical guidance due to dynamical repositioning of the laser spot. The filled red circle indicates the current or final position of laser spot. The hollow circle indicates the former position of the laser spot. Scale bar: 10 µm. (d) Kinetics of axonal turning during the multi-stage axonal turning process. Red lightning bolt indicates time at which laser spot was repositioned. (e) Growth and turning angle rates correlation histogram. Black arrow indicates time at which laser spot was repositioned.

Long-range (both angle and relative distance) axonal guidance could be realized by effecting multi-staged turns (Movie S1). By dynamically repositioning the laser spot in front of an already-deviated axonal growth cone path, multi-stage optical guidance events were observed to produce an overall turning angle of up to 120 degrees and total guidance distance of ∼90 µm (Fig. 3 a to c). Following 50 min of laser spot interaction (turning angle of ∼55°), the laser spot was repositioned, as shown in Fig. 3b. Fig. 3d shows the kinetics of axon's turning-angle during this multi-stage axonal turning process. The growth rate was found to be low during the initial phase (10 min) of interaction, but was observed to increase after 20 min (once the growth cone had turned completely). Fig. 3e shows the correlation histogram for growth rates and turning angle rates at different time points. From this single multi-stage guidance experiment, no obvious correlation between growth and turning rates can be determined. The growth per frame and total axonal growth during the long-range guidance process is shown in Figure S4c in File S1 and Figure S4d in File S1, respectively.

### Proposed mechanisms for laser spot acting as a repulsive axonal guidance cue

To test if the repulsive reaction of axonal growth cone is due to denaturing of laminin on the substrate, we coated the glass coverslips with laminin, which was fluorescently labeled by antibody. Irradiating the surface with power (100 mW, 785 nm, CW) and exposure period (30 min) did not lead to decrease (beyond photobleaching) in fluorescence intensity, indicating that the substrate was not damaged or significantly modified during the guidance experiments. The mechanism of repulsive guidance of axon due to laser can be analyzed based on three plausible effects, namely photo-physical, photo-thermal effects (leading to indirect chemical changes) or direct photo-chemical changes which occur during filopodia-laser spot interaction. First, a laser microbeam of Gaussian (or similar) cross-sectional intensity profile is known to act as a force gradient, which attracts or pulls objects toward the center of the beam [34]. Stretch-activated (or mechano-sensitive) ion channels are present in virtually all cells [35]. They serve as a primary mechanical transducer, with the channel's state changing as an immediate effect of mechanical force pressure, or pull [36]. Therefore, any sufficient mechanical force gradient could activate these mechanosensitive channels, resulting in an influx of calcium and other ions present in extracellular media. An influx of extracellular calcium is known [37] to lead to local depolymerization of axonal filopodia/growth cone and, ultimately, the arrestment of the filopodia's growth (or retraction into the growth cone). To evaluate the effect of radiation pressure forces [38] due to laser spot, simulation of the gradient force acting on single filopodia was carried out. Full derivation and theoretical treatment can be found in supplementary methods.

The filopodium (radius: a, length: l) in the instantaneous electric field of 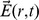 acts as a dielectric right-circular cylinder, whose dipole moment in MKS units is given by
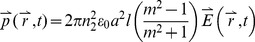
(1)where *m = n_1_/n_2_* is the relative refractive index of the filopodium and 
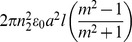
 is the so-called polarizability of a right circular cylinder, assuming that the orientation of the electric field is perpendicular to the axial direction of the cylinder. There are no closed-form solutions for a dielectric right circular cylinder without this assumption, and the problem must either be solved numerically since circular cylinders are objects with uniaxially anisotropic polarizability.

The gradient force is due to the Lorentz force acting on the dipole, which is induced by the electromagnetic field. By using the electric dipole moment, an instantaneous gradient force can be described as
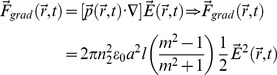
(2)The gradient force which the filopodium experiences in a steady state is the time-average version and is given by
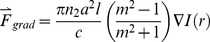
(3)By substitution, the component of the gradient force which acts along the axis of the cylinder is found to be
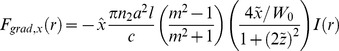
(4)


**Figure 4:**
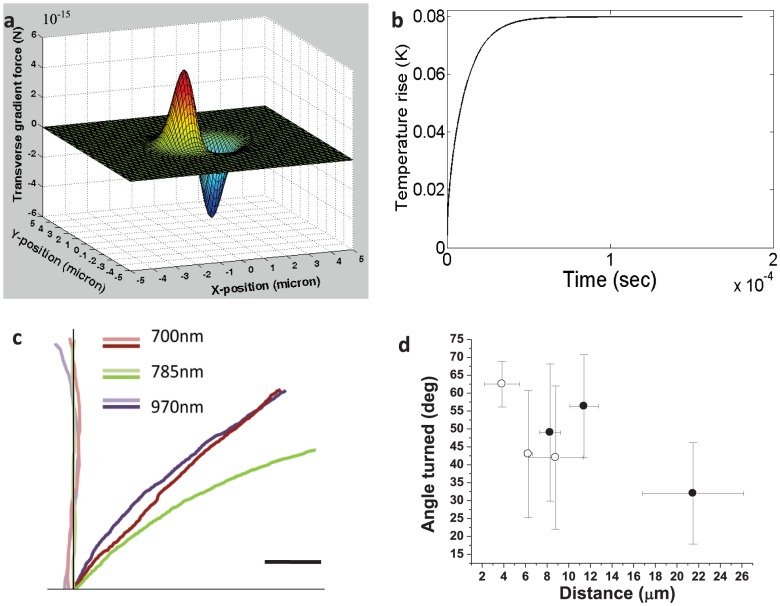
Mechanism of optically-repulsive axonal guidance. (a) Simulation of optical transverse gradient force on the tip of filopodia (diameter: 50 nm, refractive index: 1.4) due to focused laser beam (785 nm) power of 50 mW. (b) Kinetics of estimated temperature rise due to 785 nm laser beam (50 mW). (c) Axonal turning due to laser beam of varying wavelengths. Before turning (faint) and after turning (dark). Scale bar: 5 µm. (d) Variation of maximum turn angle as a function of initial distance between laser spot and tip of filopodia (open circles) or leading edge of lamellipodia (closed circle).

The simulated transverse optical gradient force on the tip of filopodia (diameter: 50 nm, refractive index: 1.4) due to focused laser beam (785 nm) power of 50 mW is shown in Fig. 4a. This force is expected to vary with laser power and interaction volume (length) of the filopodium with the laser spot. However, beyond a certain threshold (30 mW), increase in laser power did not lead to any significant increase in turning angle or efficiency. Furthermore, the axons could be guided with weakly focused (20×, NA = 0.5) laser beam, where gradient forces decrease substantially. Therefore, the role of photo-physical forces in the repulsive optical guidance of axons can be neglected.

In order to estimate temperature rise caused by the laser heating of growth medium, simulations were conducted at different wavelengths on the basis of the Fourier heat equation [39],
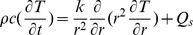
(5)where *T* is the temperature (K), *ρ* is the density (kg/m^3^), *c* is the specific heat capacity of the medium (J kg^−1^ K^−1^), *k* is the thermal conductivity of the medium (W m^−1^ K^−1^), *t* is the time (s), *Q_s_* is the heat-source term (W/m^3^) due to the heating by the continuous wave laser excitation and *r* is the radial distance (m) from the focused spot. We assumed that 100% of the energy absorbed by the medium is transferred to the surroundings as heat. Using Beer–Lambert's law: A = −log (1-*Q_s_*/*Q*) = εc*l*, where A: absorbance of the medium. Q is the incident laser power per unit volume (W/m^3^) in the focal spot, ε: extinction coefficient, *l*: path length), *Q_s_* becomes equal to the laser power absorbed by the medium per unit volume;
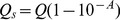
(6)


The heating control volume (m^3^) for the calculation was based on the radius of focal spot (*R = *0.4 µm) and path length *l* (depth of focus = 2π*R* ^2^/λ) of 1 µm. The heat transport equation was solved by an explicit finite-difference method given by
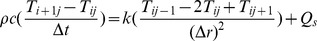
(7)where Δ*t* and Δ*r* are the characteristic time and distance steps, and *i* and *j* are the corresponding indices. Full theoretical treatment of temperature rise kinetics is shown in supplementary methods.

Fig. 4b shows the kinetics of estimated temperature rise in the immediate vicinity of the center of the laser (785 nm, 50 mW) spot, by considering water as the only absorber (absorbance; 3×10^−2^ cm^−1^). Another two near-infrared wavelengths (700 and 970 nm) were simulated (Figure S5 in File S1), as absorbance (A) of the medium (water) is quite different at these two wavelengths (5×10^−3^, and 2×10^−1^ cm^−1^ for λ = 700, and 970 nm respectively). Absorption of the laser energy directly by the filopodium was neglected due to its extremely small thickness and perceived large distance (>2 µm, in experimental settings) from the laser spot. Specific heat (*c*) and thermal conductivity *(k)* of water (4200 J kg^−1^ K^−1^ and 0.58 W m^−1^ K^−1^ respectively) was used for these simulations. The density of the medium was also assumed same as water, *ρ* = 1000 kg m^−3^. As shown in Fig. 4b and Figure S5 in File S1, the temperature rise in the center of laser spot was found to increase with laser irradiation time. At laser power of 50 mW, the saturation temperature rise was estimated to be 0.01, 0.08 and 0.5 K for irradiation wavelengths of 700, 785 and 970 nm respectively. It may be noted that experimentally temperature rise of ∼1 K/100 mW has been measured [40] at 1064 nm. The saturated estimated peak temperature rise was found to increase linearly with laser beam power. Though temperature rise can affect polymerization of actin directly [41], we believe it is the indirect photothermally-driven chemical process which is dominant in our guidance scheme. The spatial distribution of temperature around the center of the focused spot showed that the temperature decreases with increasing distance from the center and levels off at ∼2 µm from the center.

Though the overall temperature rise may seem very low, a temperature gradient of ∼0.005 to 0.25 K/µm (i.e. 5 to 250 K/mm) is estimated for the laser wavelengths (700–970 nm) and power level (50 mW) used in our experiments. Further, our simulations represent a lower limit of the heating effects, as we have neglected possible higher absorbance of medium relative to that of pure water. It is plausible that growth cone (filopodia) can sense such temperature gradients and therefore guide the axon in response to the light beam's temperature effect. Membrane ion channels which are sensitive to both temperature and mechanical stretching are known to be present. For experimental evaluation of the possibility that the turning phenomenon is induced by localized heating effects caused by media's absorption of the laser beam, trials were conducted with λ = 700, 785, and 970 nm. In spite of the fact that the temperature gradients created by 700 and 970 nm laser beams vary widely, no statistically significant difference in total angle of axonal turning was observed. Fig. 4c shows the initial and final traces of growth cone plus 10 µm length of axonal shaft for individual guidance experiments at 700, 785, and 970 nm. It is therefore possible that, even at 700 nm, the associated temperature gradient at 50 mW sample-site power is steep enough to induce repulsion of the advancing growth cone and that this phenomenon saturates with increasing gradients.

In order to further evaluate the role of photothermal effects, axonal turning experiments were conducted at varied distance of the laser spot from the filopodium, keeping the laser power fixed. Fig. 4d shows variation of maximum turning angle as a function of initial distance of filopodium (open circles) from the center of the laser spot. Also shown is the turning angle variation with separation of leading edge of lamellopodium (filled circles in Fig. 4d) from the laser spot. While there is a sharp decrease in turning-angle with increase in filopodium-spot distance, the optimal turning angle-distance is still found to be ∼3 µm away from the laser spot. This action-at-a-distance would indicate involvement of photothermal mechanism and rule out mechanical force or direct photochemical-based interaction between laser spot and filopodium. When the laser spot was initially positioned on the filopodium (and/or lamellopodium), the growth cone was found to retract and terminate (in majority of cases), indicating axonal damage. Therefore, we postulate that filopodia responds to the laser-induced thermal gradient leading to the observed repulsive optical guidance.

It is known that the neuronal growth cone uses surface receptors to integrate the environment and accordingly navigate the axon. Cytoplasmic Ca^2+^ signals have been known [37] to play a key role in this process. The Ca^2+^ regulated motility [42], [43] has been observed to be bimodal in nature, with steep gradients of Ca^2+^ signals mediating attraction and weak gradient of Ca^2+^ signals leading to repulsion. Indeed, earlier studies have shown that lamellipodia extension decreases for laser spots with λ≥1200 nm. This can be attributed to the fact that absorption of water at 1200 nm is an order of magnitude larger than 1064 nm and it is possible that use of 1200 nm heats the medium (and cell) sufficiently to cause massive membrane depolarization and large Ca^2+^ influx, which can depolymerize the F-actins leading to retraction. In our case, the repulsive nature observed in all laser-growth cone interaction lead us to postulate that the temperature rise due to the laser beam leads to gradient of Ca^2+^ signals, which in turn steers the axon away from the laser spot. This constant, local depolymerization leads to a filopodial growth coma in the area of the laser-cell interaction while the rest of the growth cone continues in its polymerization cycle. This directly serves as a guidance cue by retarding local filopodia growth, allowing other filopodia to control the direction of axonal growth. It has been shown [44] that activity of RGCs responds to temperature changes and RGCs are known [45] to express transient receptor potential vanilloid (TRPV) channels. The TRPV channels are highly sensitive to temperature [46], and essentially serve as cellular membrane “thermometers”. While large temperatures are sensed by activation of TRPV1 & 2 channels, small temperature rise can be detected by TRPV3 and TRPV4 channels. While the mechanism of this optical navigation process will require further investigation, to our knowledge, this is the first successful repulsive optical navigation of axons over large distance and angle. The axonal guidance method reported here can serve as a mean to construct neural circuits.

If this method can be successfully demonstrated using fiber optics or weakly-focused optics, we would have the necessary working distance to influence guidance of regenerating axons toward conduits. It may be noted that current intervention strategies for spinal cord injury [47] (SCI) do not ensure robust axonal outgrowth, specifically corticospinal tract (CST), past the distal glial scar. The optical repulsive cue provides a unique opportunity for detouring the regenerating CST axons toward spared axonal tracks across the glial scar *in-vivo*. Use of such optical non-contact and non-invasive methods will significantly impact on development of innovative SCI treatments.

## Conclusions

We have demonstrated a novel laser-based method for the efficient guidance of neurons which does not require optical tweezers or microfluidic flow. This method was successful for both permanent short and long-range axonal guidance, as we observed guidance of not only the lamellipodial extension, but of the axonal shaft as well. This non-contact method permits multiple and sequential operations to be performed on a single growth cone. We have also demonstrated that both laser spot and spatially-sculpted line beam results in statistically significant growth cone turning, independent of operational wavelength. The optimization of effective laser profiles, as well as the integration of automated laser/stage systems should allow for dynamical control of axonal growth paths. Further, by using high speed spatial light modulator and several laser beams, it should be possible to create multiple, spatially-sculpted light spots in order to allow simultaneous control of one or more growth cones. It is important to note that the proposed mechanism behind this method of optical-based axonal guidance does not rely exclusively on a tightly focused laser beam (i.e. optical tweezers), and is therefore potentially realizable using optical fibers. As a fiber optic technique, this method of axonal guidance has potential for future clinical and research applications.

## Supporting Information

File S1 Figure S1 in File S1: Optically-repulsive axonal guidance method. (a) Schematic of the laser axonal guidance setup, (b) Pseudo-colored time-lapse overlay of optically-guided axon. Green cutout represents initial orientation. Red overlay is final orientation. Figure S2 in File S1: Time-lapse images of a sham experiment conducted using phantom laser spot (white circle). All sham trials resulted in growth cone passing through phantom laser spot, with negligible growth cone turning. Figure S3 in File S1: Kinetics of optically-repulsive axonal guidance. (a) Overlay of time-lapse images of axonal shaft turning toward right, (b) Kinetics of axonal right-turning. Figure S4 in File S1: Effectiveness of optically-repulsive axonal guidance. (a) Cumulative distribution. The angles varied from −90° to −90°, with the positive and negative value indicating that the axon was turned toward the left -and right-side of the laser spot, respectively. (b) Growth kinetics of an axon for single interaction. (c) Growth per frame of an axon during multi-stage turning, and (d) total growth during the long-range guidance. The time point of second laser spot irradiation is marked by red arrows (c & d). Figure S5 in File S1: Kinetics of estimated temperature rise at varying wavelengths of a focused laser beam (50 mW). (a) 970 nm, (b) 700 nm.
(DOCX)
