# Supplementary material for: Highly Effective Photonic Cue for Repulsive Axonal Guidance
Source: PLoS One. 2014 Apr 9;9(4):e86292. doi: 10.1371/journal.pone.0086292 (PMC3981697; doi:10.1371/journal.pone.0086292)
Supplement: File S1 — Figure S1 in File S1: Optically-repulsive axonal guidance method. (a) Schematic of the laser axonal guidance setup, (b) Pseudo-colored time-lapse overlay of optically-guided axon. Green cutout represents initial orientation. Red overlay is final orientation. Figure S2 in File S1: Time-lapse images of a sham experiment conducted using phantom laser spot (white circle). All sham trials resulted in growth cone passing through phantom laser spot, with negligible growth cone turning. Figure S3 in File S1: Kinetics of optically-repulsive axonal guidance. (a) Overlay of time-lapse images of axonal shaft turning toward right, (b) Kinetics of axonal right-turning. Figure S4 in File S1: Effectiveness of optically-repulsive axonal guidance. (a) Cumulative distribution. The angles varied from −90° to −90°, with the positive and negative value indicating that the axon was turned toward the left -and right-side of the laser spot, respectively. (b) Growth kinetics of an axon for single interaction. (c) Growth per frame of an axon during multi-stage turning, and (d) total growth during the long-range guidance. The time point of second laser spot irradiation is marked by red arrows (c & d). Figure S5 in File S1: Kinetics of estimated temperature rise at varying wavelengths of a focused laser beam (50 mW). (a) 970 nm, (b) 700 nm. (DOCX) [file pone.0086292.s001.docx]

**Supporting Information**

***Set up and method for axonal navigation***

The schematic of the microscopic platform for axonal navigation experiments is shown in Figure S1a. For axonal guidance, the laser spot is placed asymmetrically ahead of the actively growing growth cone (Figure S1b).

**(a)**

| **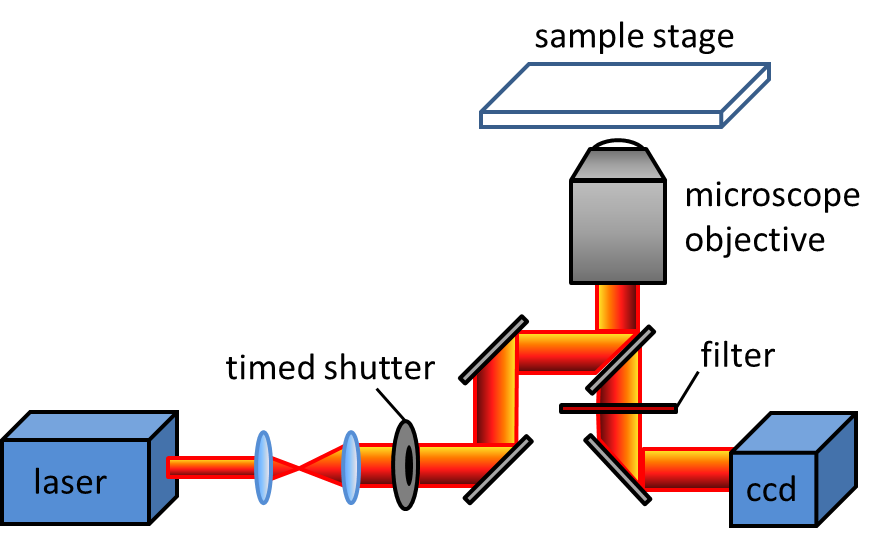** |
| --- |
| **(b)** 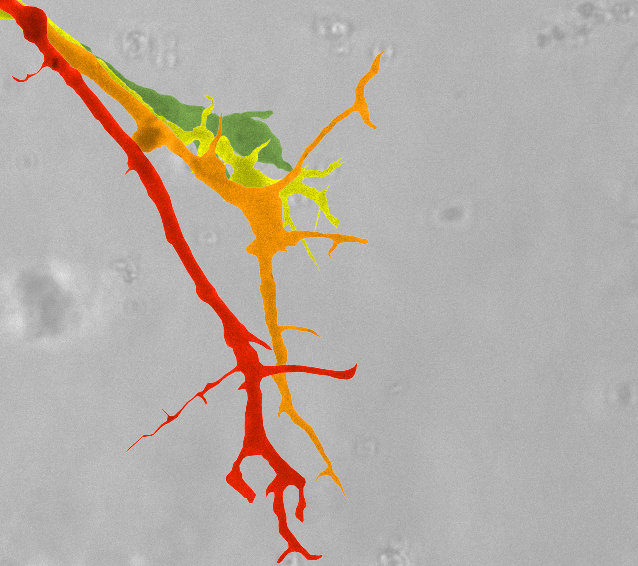 |

**Figure S1:** Optically-repulsive axonal guidance method. (a) Schematic of the laser axonal guidance setup, (b) Pseudo-colored time-lapse overlay of optically-guided axon. Green cutout represents initial orientation. Red overlay is final orientation.

Figure S2 shows time-lapse sequence of a sham experiment conducted using phantom (no) laser spot. All sham trials resulted in growth cone passing through phantom laser spot, with negligible growth cone turning.

| 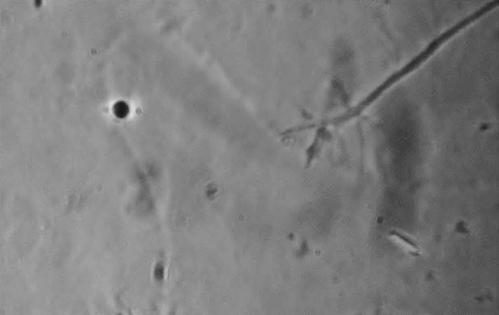  **c**  **a**  0 min | 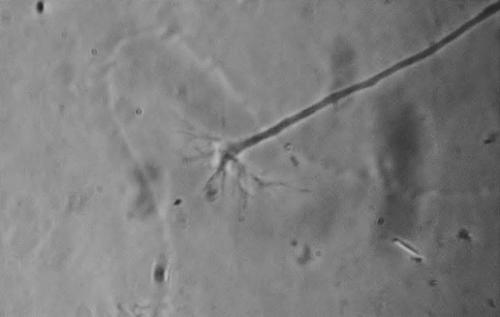  **d**  **b**  20 min |
| --- | --- |
| 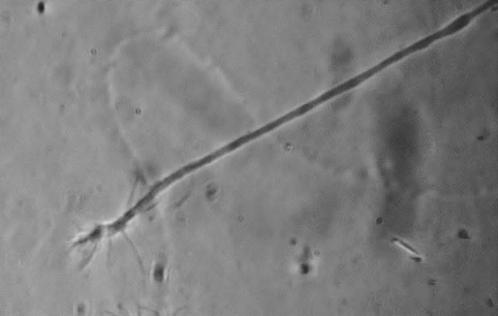  40 min | 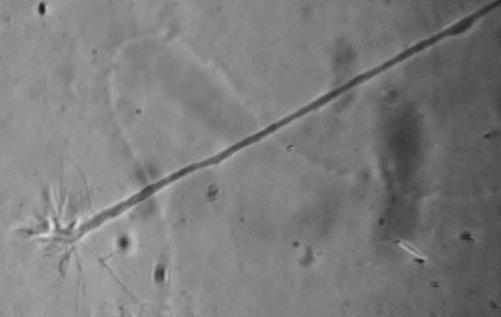  50 min |

**Figure S2:** Time-lapse images of a sham experiment conducted using phantom laser spot (white circle).

Figure S3a shows overlay of time-lapse images of axonal shaft turning toward right. The kinetics of axonal shaft turning toward right is shown in Figure S3b.

**(b)**

**Time increasing**

**(a)**

| **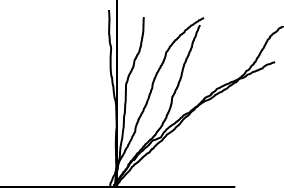** |  |
| --- | --- |

**Figure S3:** Kinetics of optically-repulsive axonal guidance. (a) Overlay of time-lapse images of axonal shaft turning toward right, (b) Kinetics of axonal right-turning.

***Kinetics of laser-assisted axonal turning.***

Figure S4a shows the cumulative distribution of turning in both left and right directions. Figure S4b shows the growth Kinetics of a typical axon. The growth rate prior to laser spot interaction did not subsequent to turning. Figure S4c and d respectively show the growth per frame and total axonal growth during the long-range guidance process.

**a**

**b**

|  |  |
| --- | --- |
| **c** | **d** |

**Figure S4:** Effectiveness of optically-repulsive axonal guidance. (a) Cumulative distribution. The angles varied from -90^0^ to –90^0^, with the positive and negative value indicating that the axon was turned toward the left -and right-side of the laser spot, respectively. (b) Growth kinetics of an axon for single interaction. (c) Growth per frame of an axon during multi-stage turning, and (d) total growth during the long-range guidance. The time point of second laser spot irradiation is marked by red arrows (c & d).

***Simulation of force on the filopodia due to laser beam***

The trapping force which acts on spherical Rayleigh objects has been derived earlier by Harada *et. al.*[1] and other researchers. We briefly describe the theoretical development of force on the filopodium as follows. Let the dielectric filopodium with radius *a* and length *l,* which has dielectric constant $\varepsilon_{1}$in a medium with a dielectric constant $\varepsilon_{2}$ and magnetic permeability $\mu_{2}$ is illuminated by a linearly polarized (II to X axis) TEM_00_ Gaussian beam with beam-waist radius W_0_ propagating to the positive Z-direction.

Let the center of the coordinate system (0, 0, 0) be located at the beam waist center and the center of the filopodia be located at $r=(x,y,z)$. Within the zero-order approximation in a paraxial Gaussian beam description, the electric-field vector at the position r is given by $\vec{E}(r)=\hat{x}\vec{E}(r)$where $\hat{x}$ is the unit vector in the polarization direction. The intensity of light at the position r(x, y, z) can be defined as a time-averaged version of the Poynting vector and is given by

$I(r)=\hat{z}\frac{n_{2}\varepsilon_{0}c}{2}|E(r)|^{2}=\hat{z}I(r),$ (1)

The intensity profile of a Gaussian (TEM_00_ mode) beam can be written as,

$I(r)=\left(\frac{2P}{\pi W_{0}^{2}}\right)\frac{1}{1+\left(2\tilde{z}\right)^{2}}\exp(-\frac{2(\tilde{x}^{2}+\tilde{y}^{2})}{1+(2\tilde{z})^{2}})$ (2)

in which $P=\pi W_{0}n_{2}\varepsilon_{0}c\frac{E_{0}^{2}}{4}$ (3)

with *E_O_* being the electric-field strength at the beam-waist center and

$\tilde{x}=\frac{x}{W_{0}}$, $\tilde{y}=\frac{y}{W_{0}}$ and $\tilde{z}=\frac{z}{\pi W_{0}^{2}}$ (4)

The filopodium (radius: a, length: l) in the instantaneous electric field of $\vec{E}(r,t)$acts as a dielectric right-circular cylinder, whose dipole moment in MKS units is given by

(5)

where *m = n_1_/n_2_* is the relative refractive index of the filopodium and $2\pi n_{2}^{2}\varepsilon_{0}a^{2}l\left(\frac{m^{2}-1}{m^{2}+1}\right)$ is the so-called polarizability of a right circular cylinder, assuming that the orientation of the electric field is perpendicular to the axial direction of the cylinder. There are no closed-form solutions for a dielectric right circular cylinder without this assumption, and the problem must either be solved numerically since circular cylinders are objects with uniaxially anisotropic polarizability.

The gradient force is due to the Lorentz force acting on the dipole, which is induced by the electromagnetic field. By using the electric dipole moment, an instantaneous gradient force can be described as

(6)

where the vector identity of $\nabla\vec{E}^{2}=2\left(\vec{E}.\nabla\right)\vec{E}+2\vec{E}x\left(\nabla x\vec{E}\right)$ have been used with $\nabla x\vec{E}=0$, as a result of the Maxwell’s equations. The gradient force which the filopodium experiences in a steady state is the time-average version and is given by

$\vec{F}_{grad}=\frac{\pi n_{2}a^{2}l}{c}\left(\frac{m^{2}-1}{m^{2}+1}\right)\nabla I\left(r\right)$ (7)

By substitution, the component of the gradient force which acts along the axis of the cylinder is found to be

(8)

***Theoretical calculation of the local temperature rise by laser***

The local temperature rise as a result of the laser heating of growth medium at different wavelengths was calculated on the basis of a numerical model. The laser-heated medium was assumed to be the only point sources of heat. Evaporation of water as a cooling mechanism was neglected. The temperature rise of medium was estimated by considering two concurrent processes: heat generation in the medium by absorption of the laser beam and heat depletion into the surrounding media by the process of conduction. Accordingly, these two processes were modeled in spherically symmetric coordinates into the Fourier heat equation [2],

$\rho c(\frac{\partial T}{\partial t})=\frac{k}{r^{2}}\frac{\partial}{\partial r}(r^{2}\frac{\partial T}{\partial r})+Q_{s}$ （9）

where *T* is the temperature (K), *ρ* is the density (kg /m^3^), *c* is the specific heat capacity of the medium (J kg^-1^ K^-1^)**,** *k* is the thermal conductivity of the medium (W m^-1^ K^-1^), *t* is the time (s), *Q_s_* is the heat-source term (W/m^3^) due to the heating by the continuous wave laser excitation and *r* is the radial distance (m) from the focused spot. Assuming that 100% of the energy absorbed by the medium is transferred to the surroundings as heat, Using Beer–Lambert’s law: A = -log (1-*Q_s_*/*Q*) = εc*l*, where A: absorbance of the medium. Q is the incident laser power per unit volume (W/m^3^) in the focal spot, ε: extinction coefficient, *l*: path length), *Q_s_* becomes equal to the laser power absorbed by the medium per unit volume;

$Q_{s}=Q(1-10^{-A})$ (10)

The heating control volume (m^3^) for the calculation was based on the radius of focal spot (*R=* 0.4μm) and path length *l* (depth of focus= 2π*R*^2^/λ) of 1 μm. The heat transport equation was solved by an explicit finite-difference method given by

$\rho c(\frac{T_{i+1j}-T_{ij}}{\Delta t})=k(\frac{T_{ij-1}-2T_{ij}+T_{ij+1}}{(\Delta r)^{2}})+Q_{s}$ (11)

where *Δt* and *Δr* are the characteristic time and distance steps, and *i* and *j* are the corresponding indices. A stability criterion[2] for the finite difference method is given by

$\Delta t\leq\frac{2\rho c(\Delta r)^{2}}{k}$ (12)

thus imposing a constraint on the value of *Δt* for a selected value of *Δr.* A decrease in *Δr* affords a higher accuracy, but at the expense of a quadratic increase in the number of time-step computations.

| **b**  **a** |
| --- |
|  |

**Figure S5:** Kinetics of estimated temperature rise at varying wavelengths of a focused laser beam (50 mW). (a) 970 nm, (b) 700 nm.

The distance step *Δr* was selected to be 100 nm whereas *Δt* was evaluated (to be 145 ns) from the limiting condition of the stability criterion. Figure S5 shows simulation results of temperature rise at two near-infrared wavelengths (700 and 970 nm), where absorbance (A) of the medium (water) is taken as 5x10^-3^, and 2x10^-1^ cm^-1^ for λ=700, and 970 nm respectively.

**Supplemental References**

1. Harada Y, Asakura T (1996) Radiation forces on a dielectric sphere in the Rayleigh scattering regime. Optics Communications 124: 529-541.

2. Huang X, Jain PK, El-Sayed IH, El-Sayed MA (2006) Determination of the Minimum Temperature Required for Selective Photothermal Destruction of Cancer Cells with the Use of Immunotargeted Gold Nanoparticles. Photochemistry and Photobiology 82: 412-417.
